# Complete Corpus Callosum Agenesis: Can It Be Mild?

**DOI:** 10.1155/2012/752751

**Published:** 2012-08-29

**Authors:** Matteo Chiappedi, Anna Fresca, Ilaria Maria Carlotta Baschenis

**Affiliations:** Don Carlo Gnocchi ONLUS Foundation, Piazzale R. Morandi 6, 20121 Milan, Italy

## Abstract

Corpus callosum agenesis is a relatively common brain malformation. It can be isolated or included in a complex alteration of brain (or sometimes even whole body) morphology. Etiology and pathogenetic mechanisms have been better understood in recent years due to the availability of more adequate animal models and the relevant progresses in developmental neurosciences. We present the case of a girl with a complete agenesis of the corpus callosum discovered at birth. She had mild learning difficulties, but reached satisfactory levels of autonomy after an individually tailored rehabilitative treatment. Her story is discussed in light of recent findings, which emphasize the possibility to exploit brain plasticity and the utility of an individually tailored approach, defined on the basis of a dialogue with the family and the patient.

## 1. Introduction

Corpus callosum agenesis (CCA) is among the most common brain malformations observed in humans [1]. Its incidence varies as a function of both diagnostic techniques and sample populations: in the general population, its estimated prevalence is 3–7 per 1000 birth, while in children with developmental disabilities it is 2-3 per 100 [2]. It is often associated with other anomalies such as Chiari II malformation (also known as proper Chiari malformation) with abnormal development of cerebellar vermis and medulla oblongata, which tend to descend into the foramen magnum, usually accompanied by myelomeningocele, basilar type encephalocele, and disorders of neural migration (which occurs concurrently in human brain development) such as schizencephaly, lissencephaly, pachygyria, and marked neuronal heterotopias. Recent neuroradiological findings [3] suggest that CCA might lie along a dysgenetic spectrum, including all commissural anomalies as part of an overall cerebral dysgenesis. The isolated form of CCA is however listed in OMIM (217990) and Orphanet (Orpha 200).

Patients with CCA have a clinical syndrome which had originally been thought to be a consequence of hemispheres' disconnection. Recent studies, however, pointed out that besides the already described abnormalities of sulcation, patients with CCA have abnormal microstructure and reduced volume of the ventral cingulum bundle, suggesting that abnormalities in intrahemispheric white-matter tracts may be an important factor [4]. Another interesting recent finding is a reduction in number of von Economo neurons, large spindle-shaped neurons localized to anterior cingulated cortex, and frontoinsular cortex in patients with CCA; this is considered another consequence of the genetic disruption that caused the agenesis [5].

Retrospective chart reviews and cross-sectional cohort studies report that 30–40% of cases have identifiable causes; however, up to 75% of cases with isolated complete CCA do not have an identified cause [2].

## 2. Case Presentation

Our patient was the fourth daughter of a married couple; no neuropsychiatric disorders were reported in the family. After a premature birth threat at 27 weeks gestational age, when maternal rest was suggested, birth was normal. Complete callosal agenesis was discovered when a MRI was performed to investigate a possible dysmorphic syndrome (since an atypical external ear aspect was evident); no other brain abnormalities were present. BAEP and flash and pattern reversal VEP were normal. EEG was normal, a part from an insufficient interhemispheric synchronization during sleep. Motor development was described by her parents as completely normal, while they reported a mild delay of phonologic development, with complete remission without any specific treatment.

We first saw this patient when she was 8 because she had learning and relational difficulties: she had insufficient reading and writing skills, and she was unable to establish a meaningful relation with peers (she used to prefer older children or adults as play partners, thus exploiting their relative maturity). She also missed many school days, due to nonorganic abdominal pain and sleeping problems (frequent nightmares). The neurologic exam was almost normal, a part from some confusion in quick differentiation between right and left hand and drawing skills not adequate for her age (she oversimplified the things she pictured). The psychiatric exam was almost normal, with a tendency to smile almost always and a sort of vacuity which were seen as symptoms of a hypomania. Her IQ was normal: in the Raven progressive matrices (colored form), she reached the 75th percentile, while in the Leiter-R, the full IQ was 99.

The neuropsychological testing demonstrated a lack of central integration with secondary insufficient results in syntactic and text comprehension, attention, drawing organization, planning, praxic skills (especially when right-left alternation or exact fine coordination was required), graphomotor skills, writing, and math.

The diagnosis was formulated after a team discussion; details of the neuropsychological evaluation are given in Table 1. The family agreed to a program including a training of impaired neuropsychological functions associated with an educational intervention aiming at teaching the girl how to better understand other people's feelings and better express her own ideas, emotions, desires, and concerns. A parent training was offered to help them to adapt to the specific developmental issues posed by this girl.

After six months, we could obtain a small improvement in terms of learning skills, while somatizations (abdominal pain) and nightmares completely disappeared. The girl was able to continue attending school, with an adjusted teaching strategy agreed with her teachers.

Three years later, she was well adjusted, with complete autonomy for everyday duties and almost sufficient academic results. She had a normal social life, with positive relationships with her peers. Her major present limitation concerned situations where a high level of integration was required to obtain a given result in a limited time (e.g., mathematical problem solving).

## 3. Discussion

Since the percentage of completely “normal” patients is decreasing in more recent studies [6], one could hypothesize that a pattern of deficits in high-order cognition and social skills has become apparent as more individuals with primary CCA but without obvious neurological deficits have been identified and assessed with sensitive standardized neuropsychological measures [7]. A long followup is, therefore, required even in apparently “benign” clinical conditions.

In general terms, three clinical patterns seem to be relatively common.

1. Severe neuropsychiatric deficit, usually seen in complex brain malformative diseases in which CCA is only one feature (and often not the most relevant one, in terms of consequent disability);
2. other neurodevelopmental diseases, including autism spectrum disorders, without a well-defined role of CCA in the etiology of the disorder;
3. apparently benign conditions, with IQ in the normal range but relevant neuropsychological deficits.

These ones include impairment of abstract reasoning, problem solving, comprehension of syntactic and linguistic pragmatics [2].

It is well known that life events (obviously including rehabilitative treatments) can change the way our brain works and even its shape, if they have appropriate intensity and time length. This is particularly true for developing brains, but a place for CNS plasticity is now recognized even for adults and aged people. This latter fact is important for the patient we presented, because she was first referred to our rehabilitation unit when 8, without any previous rehabilitative intervention.

The brain's complexity arises from its connectivity as is highlighted by the disproportionate increase in white matter volume throughout primate evolution [8]. Since the corpus callosum, with over 190 million axons, is the largest structure connecting the two cerebral hemispheres [9], its importance is self-evident.

Experience can modify established functional patterns. This has been shown to be true for instance for intermanual interactions [10]: rehabilitative treatment can be seen as an occasion to offer an experience which might in the long run modify neurological pathways through CNS plasticity. Studying sound lateralization in subjects with callosotomy, callosal agenesis, or hemispherectomy, some authors have suggested that “congenital absence of the corpus callosum may result in processes of neural plasticity that compensate for the reduced transfer of auditory spatial information between the cortical hemispheres” [11]. It should also be considered that the corpus callosum reaches a size comparable to the adult one by 2 years of age, but it is one of the last systems to complete myelination, a process which starts at the fourth month of pregnancy but continues into adulthood [12].

An important rehabilitative target is therefore the possibility to exploit CNS plasticity: even if an important structure such as the corpus callosum is lacking, we can offer a specific exercise and a modified environment to improve functional and structural brain adaptation. In this way, a spontaneous compensation through CNS plasticity can be enhanced, so that more complex stimuli and situations can be correctly processed and/or processing time can be reduced without losing accuracy. This rehabilitative target can be obtained through a specific training and using an adequate setting.

CNS plasticity however is likely to be insufficient to compensate any neuropsychological deficit in any possible situation, as for instance suggested for synchronization of multimodal lateralized information [13]. This implies that another relevant rehabilitative goal is the possibility to detect those situation where deficits can lead to a significant impairment and to activate helpful strategies (e.g., asking for explanations when a metaphor is used, given the high possibility to misunderstand its meaning). Interestingly, it is possible that this could lead to an improvement of the neuropsychological functioning, activating brain plasticity that leads to a nonstandard use of cerebral areas (as in the case of language [14]).

Parents of individuals with CCA and relatively normal neuropsychiatric conditions consistently describe impaired social skills and poor personal insight as the features that interfere most prominently with the daily lives of their children. Specific traits include emotional immaturity, lack of introspection, impaired social competence, general deficits in social judgement and planning, and poor communication of emotions [2]. Consequently, these patients often have impoverished and superficial relationships, suffer social isolation, and have interpersonal conflict both at home and at school due to misinterpretation of social clues: tendency for deficient social cognition in individuals with ACC seems to stem from a combination of difficulties in integrating information from multiple sources (e.g., verbal and visual ones), in using paralinguistic cues for emotion, and in understanding nonliteral speech [15]. This has been hypothesized to be the consequence of largely intact right hemisphere mechanisms supporting psychophysiological emotional response without having the possibility to integrate the intact mechanisms of the left hemisphere, due to the lack of communication and perhaps to a dysfunction of the anterior cingulated cortex [16]. This problems with emotional arousal can be the cognitive substrate for the psychopathological feature known as “alexithymia” (i.e., “lack of words to describe emotions”). A conduct disordered behavior can stem from the inability to appropriately respond to complex demands, particularly under conditions of high stimulation, in a way somewhat similar to what might happen in psychosomatic disorders [17].

This implies that a counseling should be offered to parents and teachers to help them finding ways to approach and help these children, without being stopped by their neuropsychological deficits but trying to overcome them with adequate strategies. Neuropsychological testing can be used as a guide to tailor the appropriate rehabilitative treatment [7], together with an attention to the specific needs of that given patient [18], and also in order to improve compliance [18].

## Figures and Tables

**Table 1:** Neuropsychological profile of the patient.

Leiter-R (full IQ)	99
Raven progressive matrices (Colored form)	21/36 (75° percentile)
Constructional praxis	
Block building models	6/8 (slightly below average)
Borrelly-Oleron	0/8 (below average; does not recognize the need of a hidden cube)
Modified Frostig test	
(i) QPVG	5° percentile
(ii) QPVMR	21° percentile
(iii) QIVM	13° percentile
Memory	
(i) Digit span	Normal
(ii) Verbal span	Normal
(iii) Corsi block-tapping test	*z* score: −2
Tower of London (executive functions)	
(i) Score	5°–10° percentile
(ii) Decision time	*z* score: −0.5
(ii) Executing time	*z* score: −0.55
(iv) Total time	*z* score: −0.67
(v) Violation of rules	6 (high)
Modified bells cancellation test	
(i) Rapidity	Below 10° percentile
(ii) Accuracy	Below 10° percentile
TROG	*z* score: −1.87
Text reading	
Rapidity/correctness	
(i) Rrapidity	*z* score: −1.4
(ii) Correctness	7 errors (average)
Comprehension	3 correct answers out of 10 (below average)
Writing	
Dictation	Unable to complete the test; however, although writing was readable, the number of errors was significantly higher than expected
Mathematic abilities	
(i) Number knowledge	Very poor
(ii) Calculation	Very poor
